# Planetary Health and Mental Health Nexus: Benefit of Environmental Management

**DOI:** 10.5334/aogh.4079

**Published:** 2023-07-24

**Authors:** Pushpam Kumar, Luke Brander, Manasi Kumar, Pim Cuijpers

**Affiliations:** 1 United Nations Environment Programme (UNEP), Kenya; 2 Institute for Environmental Studies, Vrije Universiteit, The Netherlands; 3 University of Nairobi, Kenya; 4 Vrije Universiteit Amsterdam, The Netherlands

**Keywords:** mental health, environment, climate change, green spaces, air pollution, cost of mental illness, human development

## Abstract

**Background:**

Human activities have induced unprecedented global shifts in natural systems including the climate, the oceans, cryosphere and biosphere. The impacts of these changes on physical health are clear and are accelerating at an alarming rate. Climate change and its consequences, especially disruptive events like floods, droughts and heat waves also impact the mental health of affected populations, increasing risk for post-traumatic stress, depression and anxiety disorders. However, the impact of climate change on mental health is not well examined and has received less attention than climate’s impacts on physical health.

**Goal:**

The paper examines the planetary health–mental health nexus. It assesses the existing state of knowledge on the association between climate events, natural disasters, pollution, access to green space and mental health. It also presents a global analysis of the economic costs of climate-related mental health disorders by developing scenarios estimating the costs of mental illness at the country level predicted to be attributable to changes in environmental factors during the period 2020–2050.

**Findings:**

Globally, the *additional* societal costs of mental disorders due to changes in climate-related hazards, air pollution and inadequate access to green space are estimated to be almost US$47 billion annually in 2030. These estimated costs will continue to grow exponentially to US$537 billion in 2050, relative to a baseline scenario in which these environmental factors remain at 2020 levels.

**Conclusions:**

Our scenario analysis shows that the costs associated with climate-related mental health morbidity and mortality are high already and continue to will increase sharply in coming decades. There is need therefore to strengthen the evidence linking climate change to mental health and to prioritize the development of evidence-based and impactful interventions to address the global burden of environment-related mental disorders.

## 1. Framing of the Research Problem

Mental health is an important component of public health. The UN Secretary General has recently been found to emphasise that “there is no health without mental health [1].” One in four people suffers from a mental-health related issue. Human health has improved dramatically at the global scale in recent decades. We have, for example, experienced a global decline in under-five mortality rates by 49% and in maternal mortality rates by around 38% between 2000–2017 [2,3]. However, broadly speaking, significant improvements in global health indices have coincided with large-scale environmental degradation [4]. The climate change is visible through heat waves, drought and flood. The shrinking of green space and loss of biodiversity and ecosystems are prevalent all over. The Intergovernmental Science-Policy Platform on Biodiversity and Ecosystem Services (IPBES) estimates that a million species are facing the risk of extinction [5]. Pollution, especially air and chemicals, is on the rise.

The human- and environmental-health nexus is critical to understand, as environmental health is an important determinant of human health [6,7], though the association rests on a relatively small empirical evidence base in formal public health research [8]. As articulated by a Rockefeller Foundation report: “we have been mortgaging the health of future generations to realise economic and development gains in the present [9].” The negative outcomes of prioritizing development over sustainable living has presented us with many challenges.

If current trends continue, anthropogenic alterations to natural systems are at great risk of triggering non-linear deleterious or even catastrophic environmental changes that pose a great threat to human civilization [10]. A triple planetary crisis – *climate change, loss of biodiversity and natural habitat, and the increase in pollution* – may have catastrophic impacts on human health [11]. The harm caused by climate change disproportionately affects the most vulnerable populations, including children, women and girls, older adults, ethnic minorities, and poorer communities [12,13,14,15]. These disadvantaged groups have a high exposure and susceptibility to disasters and also a lower ability to cope and recover from the damage suffered [16].

The association between environmental factors and physical illnesses, such as cardiovascular disease, chronic communicable diseases and non-communicable diseases, has generally received more attention than mental illness. It is widely recognised, however, that going forward, discussions about the natural environment and health must include mental health. There should be no doubt that climate events, such as flooding, tornadoes and landslides are direct causes of increased levels of post-traumatic stress disorders, depression and anxiety, although the extent of this impact is not known. It is also possible that pollution and an increasing lack of green spaces are related to increased levels of mental disorders, although a direct link cannot be assumed as is the case for climate events.

A recent review describes the ways in which mental health may be affected by climate change via four distinct pathways [17]. First, discrete events, such as natural disasters may have a direct impact on mental health. Floods, heatwaves, tornados and hurricanes, wildfires and earthquakes may be associated with increased rates of post-traumatic stress and depression, substance use disorders, suicidal thoughts and gender-specific risk factors, such as domestic abuse [18,19,20,21].

Second, mental health can be affected by gradual changes, such as rising sea levels and higher temperatures. Although the causal mechanisms are not clear, increased temperatures, for example, have been associated with more aggression and higher suicide rates [17].

Third, climate change may affect existing physical and social systems, and these changes may have indirect impacts upon the mental health of individuals and communities at large. For example, occupational structure and agricultural conditions may dramatically change in rural communities, resulting in economic uncertainties for some groups. Migration may also be driven by regions becoming less habitable or disappearing altogether (due to sea-level rise), as was the case for the residents of the Isle de Jean Charles off the coast of Louisiana, which became the first recognised climate refugees in the United States [17]. Forced migration due to ecological collapse, natural disasters and inundation of land has been increasingly observed in the Global South as well, in Pacific Islands like Tuvalu [22], and Central America, where climate-change driven disasters have caused forced displacement [23]. These disasters have increased in the current engagement.

The fourth pathway refers to the perception of climate change. There are reports of “climate anxiety” or “eco-anxiety” in parents who are worried about their children’s future and in young adults who are reluctant to procreate because of their fears about the future [17]. Solastalgia, the distress caused by the transformation and degradation of one’s home environment, is a comparable phenomenon that is so far better understood than eco-anxiety, although there is still ambiguity around how it affects mental health [24]. Climate change has also been explicitly identified as a social and environmental determinant of health in children and adolescents. Concerns have especially been related to large-scale climate events in that they can trigger distress in children and adolescents with pre-existing mental illnesses. Furthermore, those lacking social support may be at elevated risk for climate change–related mental health effects [25,26]. Multilevel risk and protective factors must be considered across family, community, sociocultural and ecological realms of existence to devise interventions and policies [27].

Mental disorders have major negative economic impacts in the form of treatment costs, loss of productivity, and impaired wellbeing, among others [28,29,30]. It has been estimated that the global economy loses more than US$1 trillion per year due to common mental disorders, such as depression and anxiety, alone [31]. Fortunately, however, the return on investment of addressing these issues is high. For every US$1 invested in improving common mental health conditions, US$4 is returned, in addition to the non-quantifiable improvements in individual lives, communities, businesses, economies, and society at large [32]. Chisholm et al [33]. estimated that scaled-up investment in mental health treatment (for depression and anxiety) was US$147 billion and can have an expected return of US$399 billion, while the cost of inaction can lead to up to 12 billion days of lost productivity (50 million years of work) and with an associated monetary loss of approximately US$925 billion.

Information on the costs and benefits of investing in mental health can be used for prioritising public health policy responses [33,34,35,36,37] and evaluating broader measures to mitigate or improve environmental determinants of mental health [38]. Thus, a closer look at the long-term economic costs, costs of action versus inaction, and investment benefits of mental health on society is urgently required. Similar to gender-related recommendations made for prior disease outbreaks, it is crucial for public health policies and interventions to recognise the extent to which mental health issues impact women and men differently and in some cases unequally, and to include this aspect in budgets to make gender-differentiated research, policy and programmatic interventions as possible.

The reduction in economic costs related to improved mental health outcomes is referred to as the “benefits of action,” the actions taken to mitigate the underlying causes of those mental health impacts. This could be, for example, the reduction in costs (to society and individuals) of the prevention and treatment of mental illness or the reduction in loss of income from mental illness. Conversely, the increased economic impacts of mental ill health attributable to unchecked causes can be interpreted as the costs of inaction. Several country-level investment case studies have conveyed same sense of urgency of action needed (see the investment cases of Philippines, Kenya, Nigeria, Uzbekistan and others) [39].

## 2. State of Knowledge on Planetary Crisis and Mental Health

Climate change, pollution and the loss of nature are global threats driven by the unsustainable practice and pace of anthropogenic activities. Applying rigorous selection criteria, detailed in Appendix 2, this synthesis of evidence identifies 24 studies for inclusion in the final review. The largest number of studies are those looking at climate change, followed by air pollution and green spaces.

Our review points to methodological and theoretical problems with many of the studies that examine the linkages between environmental degradation and mental health. There is work to be done to improve the measurement, identification of key variables, and design of conceptual models to provide high-quality evidence on mental illness prevalence and environmental stressors. Correlation observed within the reviews does not imply causality, and given the complex nature of the underlying processes, we cannot unequivocally say that those are the only influences on mental health. This limitation continue to be a significant barrier in creating new knowledge around mental health and environmental stressors.

Very few studies from low- and middle-income countries exist on these issues. In general, mental health epidemiologic evidence comes mostly from high-income countries where these problems are more extensively researched and established systems for assessing mental health exist. Very often, the environmental footprints in low- and middle-income countries continue to be more damming due to the development priorities. We must also remember that the socio-economic gradient is critical in mental health, as poor life conditions and adversities increase the propensity for mental illnesses, aside from the psychological debilitation that might occur after exposure to extreme environmental events. Addressing the mental health needs of vulnerable sub-sections of populations, such as internally displaced, refugees, indigenous peoples, small-scale farmers, women, youth and the disabled, will be critical.

First, improvements to multiple forms of environmental conditions are opportunities to invest in mental health. Second, we highlight that effort is needed to strengthen research and expertise in understanding the pathways between environmental stressors and mental illness in order to develop interventions that seek to promote mental health, including collective action, policy and legislative tools. To this end, the analysis presented in the next sections of this report applies an economics lens to estimate the costs of mental illness attributable to changes in environmental factors.

This nexus of environmental challenges impacts the economic wellbeing and health of all human societies, including their constituents’ mental health. Environmental degradation and the mental health of human populace are both complex phenomena that we have not yet sufficiently invested in to better understand their drivers, consequences, and interlinkages. The relationship between environmental decline and mental health requires deep epistemic thinking and development of conceptual models that speaks to intervening, moderating and mediating variables which could spread across time, space and forms of life.

## 3. Methodology for the Explorative Economic Analysis

To estimate the country-level costs of mental disorders attributable to changes in environmental factors we use value transfer methods, which involve using existing primary valuation studies at one or more *study sites* to estimate values for similar locations or *policy sites* [40,41]. Value transfer methods are widely employed for estimating economic values of environmental impacts in national and global assessments [42,43] but can be inaccurate due to differences in the bio-physical and socio-economic contexts of study and policy sites [44]. For applications at large geographic scales covering multiple diverse policy sites, the use of value functions estimated through regression analysis of primary valuations offers a means to systematically adjust transferred values to reflect the variation in factors that influence the economic value of environmental impacts [45,46,47]. The motivation for applying value function transfer methods in the present study is to facilitate the global scale of analysis, which would be unfeasible using primary research methods, and to enable consistency in the estimation of values across countries.

**Figure 1:**
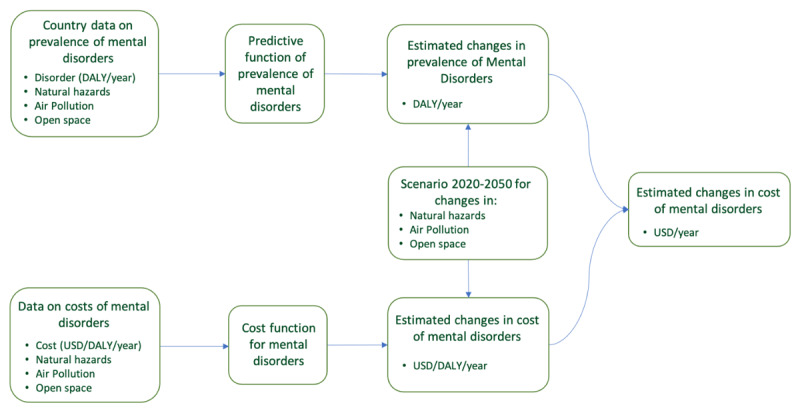
Methodological framework to explore the economic relationships between the prevalence and costs of mental health and selected environmental factors.

The methodology applied in the explorative economic analysis of the relationships between (1) the *prevalence* of mental health and (2) the *costs* of mental health and selected environmental factors is represented in Figure 1. The study combines data and models from several sources. Two regression analyses were used to quantify the relationships between mental health and three environmental factors: (1) natural hazards, (2) air pollution, and (3) access to open (green) space.

The first analysis is conducted at the country level to attempt to explain **variation in the prevalence of mental health disability-adjusted life years** (DALYs)^1^ as a function of the environmental factors (see Section 4).

The second analysis examines relationships between **the costs of mental health** (USD/DALY) and the environmental factors (see Section 5).

The estimated regression models are subsequently used in a scenario analysis to quantify **changes in the prevalence of mental illness and their societal costs** for the period 2020–2050 (see Section 6).

### 3.1 Analysis of prevalence of mental illness and environmental factors

#### 3.1.1 Methodology

A multivariate regression analysis is used to quantify relationships at the country level between the prevalence (rates) of mental illness, the set of environmental factors, and income level and inequality. The data for this analysis are obtained from a variety of sources (Table 1). The principal source of data on rates of mental illness is the Global Burden of Disease Initiative [48], which provides information on the prevalence of diseases and the relative harm they cause for the period 1990–2019 for 204 countries. We measure the change in number of mental health DALYs per 100,000 people in 2019.^2^,^3^

**Table 1:** Definitions and sources of data used in the country level analysis.


VARIABLE	DEFINITION	SOURCE	MEAN	S.D.

DALY rate	Number of mental health DALYs per 100,000 people 2019	Global Burden of Disease	1,689.59	328.27

GDP per capita	Gross Domestic Product per capita 2019 (USD; thousands)	World Bank	17.34	24.04

Inequality	Index of inequality (0–10)	INFORM Risk	4.04	1.95

Healthcare inaccessibility	Index of inaccessibility to healthcare (0–10)	INFORM Risk	4.13	2.45

Hazard exposure	Composite index comprising data on hazards, exposure, vulnerability and coping capacity (0–10)	INFORM Risk	3.75	1.77

Air pollution	PM_2.5_ mean annual exposure (micrograms per cubic meter)	World Bank	27.08	18.83

Access to green space	Proportion of population living in urban areas (%)	World Bank	60.56	23.41


See Appendix 1, Table A1.1 for further information.

Extreme events put significant stress on people’s physical and mental health, manifesting as, for example, post-traumatic stress disorder, depression and anxiety, as discussed in Section 1. We use the Hazard Exposure Index as a proxy for extreme natural disasters and climate change events. The Hazard Exposure Index is a comprehensive index ranging from 1 to 10. It accounts for the exposure to natural and human-induced hazards, socio-economic vulnerability, vulnerable groups, and coping capacity [49].

In analysing the association between pollution and mental health, especially depression, suicide and autism spectrum disorders, as presented in Section 2.3.2, Pollution and Mental Health, we use PM_2.5_ mean annual exposure as the measure of air pollution [50].

We use the proportion of the population living in urban areas as a somewhat crude proxy for access to green space. This will allow analysis of the association between green spaces and depression noted in Section 1, with the assumption that a higher proportion of the population in urban areas have less access to green space and natural environments compared in rural areas.

We control for variation across countries in terms of income, inequality and access to healthcare. For income, we use gross domestic product (GDP) per capita in US$ in 2019 prices for the country-level assessment and US$ in 2020 prices [50]. To explicitly account for inequality effects, we include a separate predictor variable that is a composite index of income and gender inequality, obtained from the INFORM initiative [51]. We use healthcare inaccessibility data to quantify access to or availability of healthcare facilities [49].

### 3.2 Results

We find positive and statistically significant relationships between exposure to natural and other hazards, exposure to PM_2.5_ and absence of access to green space and the rate of mental illness (Table 2). These results provide empirical quantification of the increase in mental illness prevalence attributable to deterioration in these environmental factors at a country level. The umbrella meta-analysis presented in Section 2 provides a critical review of the existing literature on these relationships and points to the need for more rigorous research to develop our understanding of the underlying processes. While documenting available evidence, this section aims to provide economic costs along with adjustments made along the socioeconomic determinants of mental health.

**Table 2:** Statistically significant relationships from regression model of rates of mental disorders as a function of environmental, economic and socio-economic factors.


	REGRESSION COEFFICIENT

GDP per capita	**3.57**

Income inequality	**38.07**

Healthcare inaccessibility	**–94.06**

Hazard exposure to extreme natural disasters	**42.68**

Air pollution	**2.12**

Access to open (green) space	**3.53**


*Note*: Dependent variable is the number of DALYs per 100,000 people. Full regression model is given in Appendix 1, Table A1.2.

We find that GDP per capita and income inequality have statistically significant positive relationships with rates of mental illness, suggesting that wealthier and more unequal societies record higher rates of mental disorders. We find that lack of access to healthcare is negatively related to mental illness rates, which indicates that healthcare systems with lower capacity diagnose fewer cases of mental disorder.

## 4. Analysis of Cost of Mental Illness and Environmental Factors

A meta-regression analysis is used to quantify the relationship between the costs of mental disorders and a set of explanatory variables including environmental factors.

### 4.1 Methodology

The principal source of cost data was Christensen et al., [30] which evaluates the results of 143 articles that estimate the costs of mental illness in monetary units. These data cover 48 countries and provide almost 2,900 cost estimates. To note, is the relatively low number of cost estimates from low- and middle-income countries. There were also relatively fewer estimates pertaining to certain types of mental disorders, for example intellectual disabilities and eating disorders.

**Figure 2:**
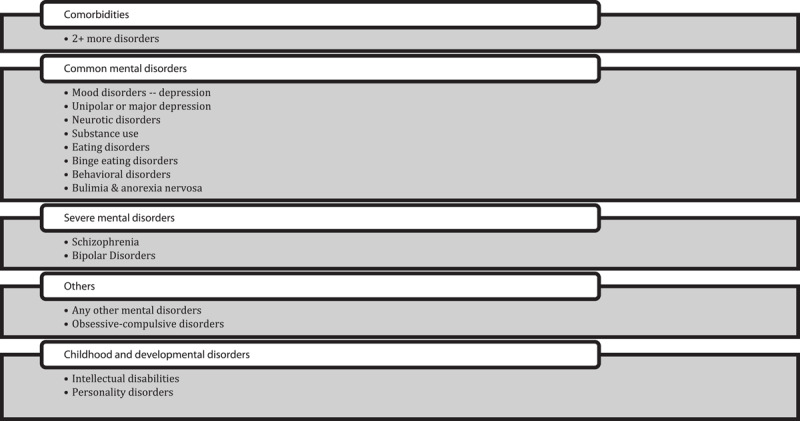
Categorisation of mental illness used to quantify the relationship between the costs of mental illness and a set of explanatory variables, including environmental factors.

We group the extensive list of mental illness and disorders provided by Christensen et al. [30] into common, severe, and childhood and developmental disorders (Figure 2). We maintained a separate cluster for 2+ disorders by comorbidities. We standardised these cost data to a common set of units, namely USD per DALY in 2020 price levels using purchasing parity adjusted (PPP) adjusted exchange rates and GDP deflators (from the World Bank), DALYs per patient (from Rehm & Shield) [52] and national DALY rates (from GBD, 2019) [48]. Our dataset contained 2,357 standardised estimates of the costs of mental disorders. These values varied substantially across the types of disorder (i.e., common, serious, childhood) and type of cost measured (i.e., healthcare, productivity loss, societal cost). Definitions of environmental variables and the source from which data was obtained for each is provided in Table 3.

**Table 3:** Definitions and sources of data used in the cost of illness analysis.


VARIABLE	DEFINITION	SOURCE	MEAN	S.D.

Cost/DALY (ln)	Cost of illness per DALY (USD; PPP adjusted; 2020 price level; natural log)	Adapted from Christensen et al. (2020)	6.50	2.61

Severe disorders	Binary variable indicating severe disorder (1 = severe; 0 = common)	Christensen et al. (2020)	0.21	0.41

Productivity costs	Binary variable indicating measure of productivity loss (1 = productivity loss; 0 = other costs)	Christensen et al. (2020)	0.09	0.29

Societal costs	Binary variable indicating measure of societal costs (1 = societal costs; 0 = other costs)	Christensen et al. (2020)	0.46	0.50

GDP per capita (ln)	GDP per capita (USD; PPP adjusted; 2020 price level; natural log)	World Bank	10.80	0.55

Hazard exposure	Composite index natural hazards (0–10)	INFORM Risk	3.36	1.79

Air pollution	Proportion of population exposed to PM_2.5_ air pollution levels exceeding WHO guideline value	World Bank	11.58	6.28

Access to green space	Proportion of population living in urban areas	World Bank	78.46	10.40


PPP = Purchasing Power Parity.

### 4.2 Results

Tables 4 and 5 present the estimated costs of mental illness by type of illness and type of cost, respectively. The data on costs of mental illness were supplemented with data for additional predictor variables: GDP per capita, hazard exposure, air pollution and access to green space. Where possible, data for the explanatory variables were extracted for the specific year in which the cost estimate was made.

**Table 4:** Cost of mental illness and development disorders, categorised by type of disorder.


	MEAN COST (US$/DALY)

Common disorders	**4,849**

Severe disorders	**11,399**

Childhood disorders	**62,071**

Childhood + development disorders	**1,120**

Total	**13,940**


*Note*: Data taken from 48 countries in Christensen et al. [30]. See Appendix 1, Table A1.3 for further statistical results.

**Table 5:** Cost of mental illness and development disorders, categorised by type of cost.


	MEAN COST (US$/DALY)

Healthcare costs	2,072

Productivity losses	9,210

Total societal costs	25,862

Other costs	7,036

Total	13,940


*Note*: Data taken from 48 countries in Christensen et al. [30]. See Appendix 1, Table A1.4 for further statistical results.

The estimated meta-regression model is presented in Table 6. As expected, we find a positive effect for severe disorders on the cost of illness, measured in US$ per DALY. The type of costs that are assessed also influence the magnitude of the costs estimated, as expected, with productivity costs and societal costs higher than healthcare costs (the omitted category in the regression model). The positive coefficient on GDP per capita indicates that the costs of illness increase with income. Regarding the environmental factors included in the model, we find a positive and statistically significant relationship between the natural hazard index and the cost of mental illness. This follows from the key findings of the umbrella meta-analysis presented in Section 2, which provides a critical review of the existing literature on these relationships, and it points to the need for more rigorous research to develop our understanding of the underlying processes. It implies that the costs of mental illness are higher in countries that are exposed to natural hazards to a greater degree. Air pollution and access to green space do not have statistically significant effects on the unit cost of mental illness.

**Table 6:** Relationships from regression model of costs of mental illness as a function of environmental, economic and socio-economic factors.


	REGRESSION COEFFICIENT

Severe disorders	**1.86**

Productivity costs	**1.76**

Societal costs	**2.56**

GDP per capita (ln)	**0.57**

Hazard exposure	**0.16**

Air pollution	–0.01

Accessibility to green space	0.00


*Note*: Dependent variable is US$ per DALY at 2020 price level. Statistically significant relationships are given in bold. Full regression model is given in Appendix 1, Table A1.5.

## 5. Scenario Analysis Quantifying Changes in the Prevalence of Mental Illness and Their Associated Societal Costs

### 5.1 Methodology

A scenario analysis is conducted here, seeking to answer the question of what the future costs of mental health would be with and without future predicted changes in climate related hazards, air pollution and access to green space. The difference in outcomes between “with” and “without” these environmental changes is understood as the additional costs attributable to the them.

The regression models presented in the preceding sections are now used to estimate the global prevalence and costs of mental illness as a function of changes in climate-related hazards, air pollution and access to green space over the period 2020–2050, with results reported for 2030 and 2050 to show medium- and long-term impacts.

The scenario analysis is conducted at the national level for 156 countries, representing 99% of the global population. Other countries, mainly small in population, are omitted from the analysis due to lack of data. Initially we estimate the number of mental health DALYs and cost per DALY for each country in 2020. In applying the cost function, parameter values are set to estimate societal costs for common mental illnesses.

To explore the impact of future changes in climate hazards, air pollution and access to green space, we obtain projected future values for these parameters in 2030 and 2050. For climate hazards we utilise the outputs from the CLIMRISK model in the form of estimated damage under an intermediate climate change scenario,^4^ expressed as percentage changes in GDP [53,54,55]. Proportionate changes in climate damage between 2020–2030 and 2020–2050 are used to compute future values for the hazard exposure index. For air pollution we use estimated future changes in air quality from anthropogenic emissions modelled using the EMAC atmospheric chemistry general circulation model [56]. Proportionate changes in the population-weighted multiple pollution index between 2020–2030 and 2020–2050 are used to compute future values of PM_2.5_ air pollution levels. For access to green space, which is proxied as the proportion of a country’s population living in urban areas, we use data on projected changes in total and urban populations from the United Nations Population Division [57]. Percentage changes in populations between 2020–2030 and 2020–2050 are used to compute future values for the proportion of the population living in urban areas.

### 5.2 Results

**Figure 3:**
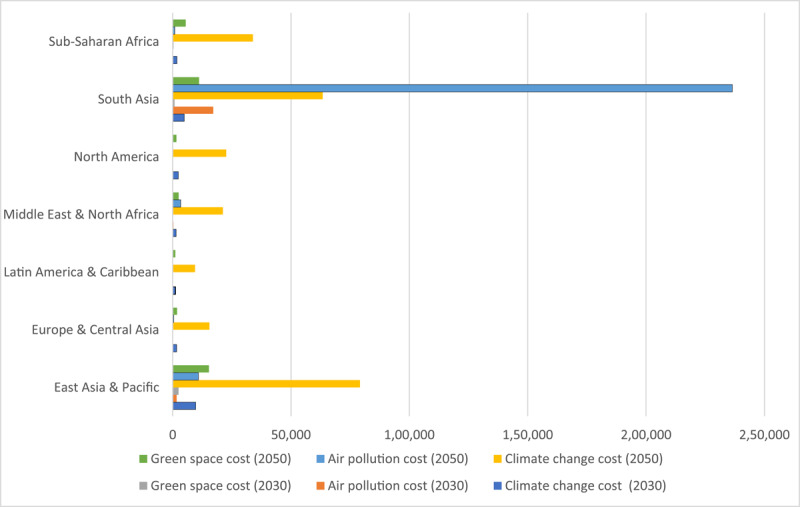
Estimated additional total annual cost of mental illness attributable to climate change, air pollution and access to green space. *Notes:* Groupings are following World Bank regional classifications.

The results are presented in Table 7 and Figure 3 per World Bank region and in Appendix 3 per country. Globally, the *additional* annual societal costs of mental illness due to changes in climate related hazards, air pollution and access to green space are anticipated to reach almost US$47 billion in 2030, relative to a baseline scenario in which these environmental factors remain at 2020 levels. The scale of additional annual societal costs of mental illness due to changes in climate related hazards, air pollution and access to green space increases substantially over time, and the costs in 2050 are an order of magnitude higher than in 2030. This reflects both worsening environmental conditions over time, leading to higher rates of mental illness, and increasing unit costs of mental illness (i.e., cost per DALY), primarily driven by increasing incomes. The global *additional* costs of mental illness due to changes in climate-related hazards, air pollution and access to green space are estimated to reach almost US$537 billion in 2050, relative to the 2020 baseline. This increase in the costs of mental illness is largely driven by climate change (US$23.2 billion) and increased air pollution (US$19.6 billion), with reduced access to green space representing a relatively small cost (US$4.4 billion).

**Table 7:** Estimated additional annual total cost of mental illness attributable to climate change, air pollution and access to green space, by World Bank region.


WORLD BANK REGION	COST(US$ MILLIONS, 2020 PRICE LEVEL)							

	2030				2050			
	
	CLIMATE CHANGE COST	AIR POLLUTION COST	GREEN SPACE COST	TOTAL COST	CLIMATE CHANGE COST	AIR POLLUTION COST	GREEN SPACE COST	TOTAL COST

East Asia & Pacific	9,739	1,666	2,490	13,896	79,128	10,919	15,318	105,366

Europe & Central Asia	1,679	54	217	1,950	15,524	476	1,851	17,851

Latin America & Caribbean	1,187	18	163	1,368	9,460	101	1,114	10,674

Middle East & North Africa	1,494	312	189	1,995	21,229	3,370	2,545	27,143

North America	2,422	11	190	2,622	22,612	85	1,598	24,295

South Asia	4,928	17,110	798	22,836	63,411	236,464	11,133	311,008

Sub-Saharan Africa	1,799	85	333	2,217	33,918	858	5,471	40,247

**Globa**l	**23,248**	**19,255**	**4,380**	**46,884**	**245,282**	**252,273**	**39,029**	**536,584**


**Figure 4:**
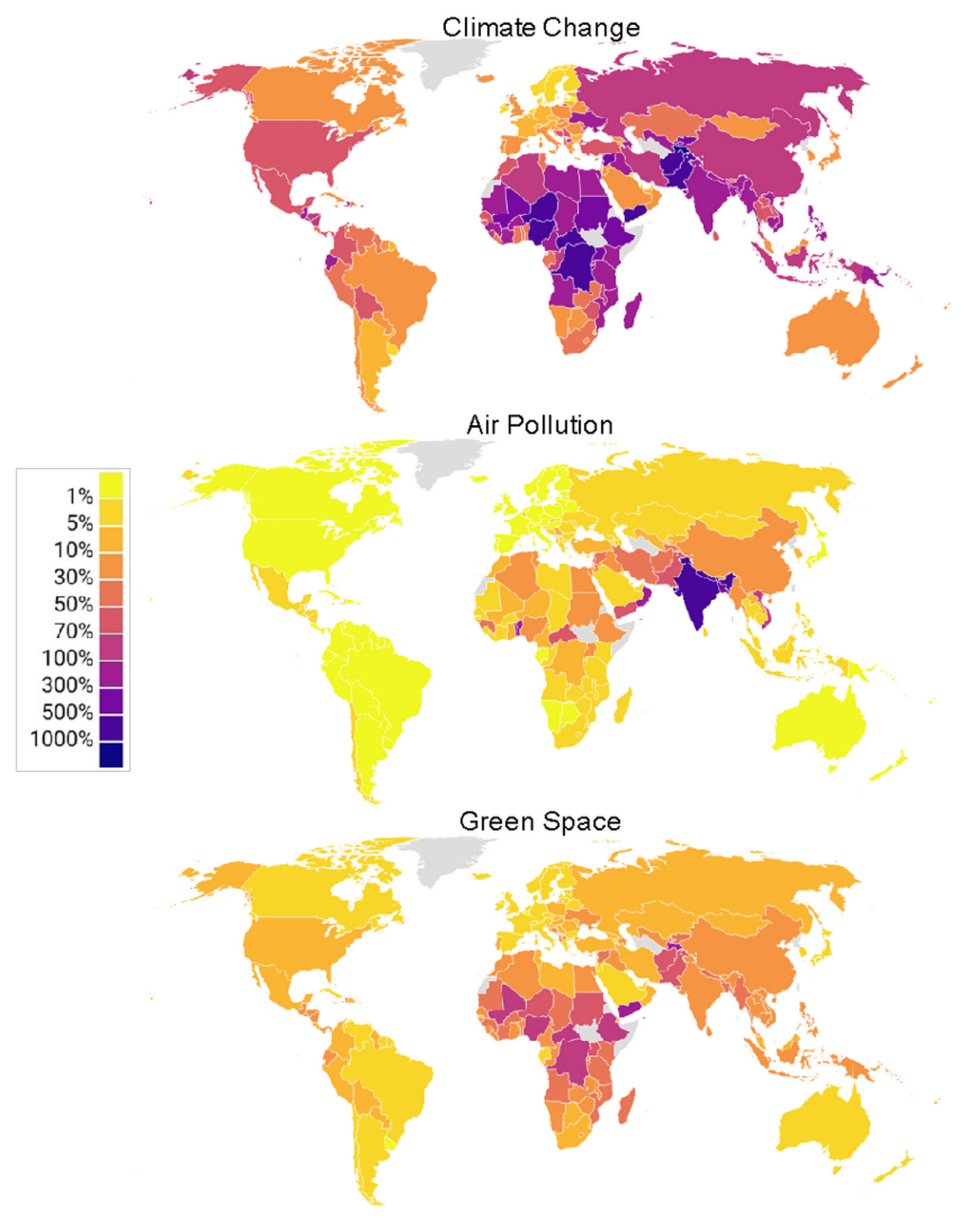
Percentage change in annual societal costs of mental illness attributable to changes in climate hazards, air pollution and access to green space in 2050 relative to 2020.

Figure 4 maps the results for 2050 in terms of the percentage change in annual societal costs of mental illness attributed to changes in climate hazard, air pollution and access to green space relative to 2020 levels. Comparison across the three maps highlights the relative impact of changes in the three environmental factors, with change in climate hazards causing substantially larger increases in the cost of mental illness.

There are, however, notable differences across countries and regions, reflecting the variations in future projections of climate change, air pollution and urbanisation. For example, South Asia faces larger increases in the costs of mental illness due to air pollution than due to climate change or access to green space; whereas in most other regions, decreasing access to green space is a larger issue than air pollution.

This can be interpreted as the mental health component of the cost of not addressing ongoing trends of environmental degradation.

## 6. Discussion and Conclusions

This study aimed to examine the association between environmental degradation and mental health. We adopted a two-stage approach: (1) a review of meta-analyses collating the state of current evidence and (2) exploratory economic analysis of the societal costs of mental health driven by climate change, air pollution and urbanization.

### 6.1 Summary of review of meta-analyses

Our review covered 24 meta-analyses, all relatively recent (post-2014), which analysed mainly cross-sectional and case-control studies.

We found some evidence that climate events have a negative impact on mental health of individuals and populations, especially manifesting as post-traumatic stress disorder, but also as depression and anxiety. We also found some evidence that pollution may affect mental health, especially depression, suicide and influencing the prevalence of autism spectrum disorders through exposure during pregnancy. The associations between some forms of pollution and mental health were significant but small. We also found some evidence suggesting a positive association between access to green spaces, and reduced risk of depression. The findings of this work has been presented in Cuijpers, Miguel, Ciharova et al. [58].

However, a number of important methodological limitations have emerged from this umbrella review on the status of research covering the impact of all three environmental factors on mental health need addressing by global research community, particularly for climate-driven extreme events. These include the following:

1. The aetiology of any one extreme weather event – distinguishing whether its cause is climate change driven or part of a natural climatic fluctuation – is difficult to ascertain. Direct attribution of climate change to mental illness is therefore difficult. To address this, the body of knowledge around the consequences (on mental health) of climate events must be increased substantially. As evidence aggregates, the consequences of multiple climate events on mental health will become clearer.
2. Even in the cases where meta-analyses in our review showed statistically valid correlations between an environmental factor and a mental health outcome, they often failed to identify causation. For example, in the meta-analyses assessing the relationship between exposure to pollution and mental health outcomes, some statistically significant, though small, correlations were found. However, although some of these studies did adjust for some confounding factors, they did not completely exclude the possibility that the association was caused by an unmeasured variable. This is even more important when the size of the identified association is small.
3. The meta-analyses further revealed that most included studies measured mental health with self-report measures, which are not sufficiently reliable to indicate the presence of a mental illness. Although studies based on mental illnesses assessed by diagnostic interviews are rare as the costs and logistic challenges are considerable, these are the gold standard of analysis as they provide statistically reliable data. There is a lot of debate within the field of mental health around measurement and assessment of mental health risk factors and psychopathology using task-shifting and self-directed assessments. These tools remain screening tools with many of these offering sound predictive validaity and psychometric properties that make these as good as a clinician led or when tested with a diagnostic tool. However cross-cultural adaptation of tools, cut-offs for different populations and tools for diverse mental health conditions are still lacking [59]. We hope that more validation and cultural contextulization of mental health measures would help fill in the measurement gap where the gold standard clinician assessment or a clinical diagnostic assessment may not be possible.
4. Finally, our review highlights that most meta-analyses examining the association between climate events and mental health not only estimated the prevalence of mental illness after the event (without a pre-event measurement), but they also pooled different measures for mental health in one meta-analysis to estimate the proportion of people affected. Critical errors such as these degrade the useability of the results for inclusion.

To establish a causal association in epidemiological research, several conditions have to be met including consistency (an association has been replicated in different settings), temporality (the cause must precede the effect), strength (a stronger association reduces the possibility for another explanation), dose-response (increase exposure leads to increased outcome), cessation of exposure (the incidence drops after cessation), probability of alternative explanations, biological or theoretical plausibility, and coherence (the association is supported by other scientific knowledge) [60,61]. For a valid conclusion to be drawn on the relationship between environmental degradation and mental health outcomes, analyses must attempt to address the conditions outlined by Gordis [61] and Pickett and Wilkinson [60] as well as address those revealed by our meta-analyses.

The limitations outlined by this umbrella review cum meta-analyses provide impetus to improving ways of thinking and developing research in mental health and environmental exposures. Capturing environmental impacts on mental health will provide key data that will enable the costs of environmental degradation to be assessed in this regard.

Current evidence provides a platform from which to develop effective policy and clinical and public health interventions. We suggest that further research addressing the limitations we outline – especially in terms of methodological rigour and quality of climate event data in reported in our published umbrella review and meta-analyses [58] – will enhance and strengthen policy recommendations. Similar findings have been reported in other reviews covering noise pollution and mental health, mental health and blue and green spaces [62]. We do acknowledge that a scoping review might have provided a further detailed explanation of relationship between environmental exposures and mental health and led to a more detailed appraisal of risk factors. However the meta-analysis has provided clear pointers to the existing evidence.

### 6.2 Explorative economic analysis of costs of mental illness

The analysis presented in this paper is characterised by several limitations and uncertainties and for this reason we describe it as explorative of the relationships between environmental degradation and mental health.

Developing a quantified measure of the impact of uncertainty on the results, such as in the form of confidence intervals, would require information on the potential (co-)distributions of underlying parameters, which are principally unknown. The analysis pools data on all mental illnesses, but it is likely that environmental determinants are more relevant to some disorders than others.

The cost-of-illness analysis is predominantly based on data pertaining to high-income developed countries. As a result, the analysis may not provide a good representation or prediction of costs for developing countries.

Access to green space is measured using the proportion of the population living in urban areas. This is a rough proxy that does not capture the substantial variation in availability of open green space within urban areas [63].

The hazard exposure index includes all sources of natural hazards, some of which are not affected by climate change (e.g., earthquakes). The projection of future changes in hazard exposure due to climate change, however, is applied to this index and may therefore over-estimate the impacts of climate change in some cases.

Future levels of exposure to PM_2.5_ are modelled using an index of multiple air pollutants, which comprises PM_2.5_ and other pollutants. All air pollutants, however, are likely to follow similar trends to PM_2.5_.

The analysis presented in this paper is at a coarse resolution using national-level data, which does not reflect large variation within individual countries. Severe disparities in mental health systems necessitate a more granular analysis of these impacts.

The analysis focuses on the potential benefits of mitigating environmental determinants of mental illness. It does not estimate the costs of mitigation options against which to compare the benefits.

Future research should seek to address these limitations of the present analysis. A key development will be to update the estimated cost function for mental illness using data from developing countries, as these become available. In terms of policy development, an assessment of the costs of mitigating or improving environmental determinants of mental health would assist in weighing up the costs and benefits of action. Applying a full cost-benefit analysis to identify investments with high net returns would, however, be challenging given the multiple ancillary benefits of environmental improvement.

### 6.3 Policy implications

The impact of environmental changes channeled through rise in temperature, shrinking of green space in urban settings, and increase in air pollution do impact constituents and determinants of mental health. The existing evidence supports this. Economic analysis, especially the quantified impact associated with loss of earning, defensive expenditure and other economic losses in human capital, helps to identify the underlying trade-offs inherent in policy decisions. This report has assessed and discussed the impact of environmental degradation (extreme weather/climate hazards, air pollution and access to green spaces) on mental health.

By incorporating the mental health costs to society of environmental degradation, both analyses comprising this report – the review and the economic analyses – have great potential to influence the development and scope of regulatory and non-regulatory action at national and local scale. The analysis in this paper serves to provide evidence to motivate policy makers to integrate environmental considerations in policy responses to address mental ill health and, concomitantly, to integrate the costs of mental ill health within broader environmental public policy debates. The climate change community and biodiversity constituency have already started recognizing this where health commands a great attention. Just concluded Conference of Parties (COPs) of the climate and biodiversity provide strong evidence need to reference the two COPs. Policy developments in mental health should consider the “leave no one behind” approach as a path for achieving the 2030 Agenda for Sustainable Development [64]. This will address inequalities (including gender and income/wealth inequalities) and provide opportunities for solving environmental conditions, mental health and their inter-connectedness in an inclusive way considering the most vulnerable and marginalized populations [65]. Applying economic analysis in public policy can ensure that policy interventions are targeted appropriately and delivered effectively. By taking into consideration age and gender-differentiated aspects as well as socio-economic factors, policy interventions are more likely to reduce incidences of mental health issues associated with environmental degradation.

The economic analysis presented here has estimated the costs to mental health from inaction in addressing environmental degradation. Not only do the models show that costs are substantial, but the economic analysis predicts that the costs of mental ill-health at the global and country level will worsen by an order of magnitude between 2020 and 2050. This will significantly burden the productivity of the workforce, as well as have substantial implications for healthcare systems and social capital.

Regulatory action on environmental degradation differs both between and within countries. Costs of inaction is likely to be disproportionately acute in low- and middle-income countries. Low- and middle-income countries are much more vulnerable than wealthier countries to the effects of climate change through extreme weather events due to a combination of factors, such as lack of resources, infrastructure, and knowledge and capacity to adequately prepare for disasters.

However, the driver of increasing frequency and intensity of extreme climate-related weather events are transboundary in nature. In effect, wealthier countries that tend to be the largest contributors to greenhouse gas emissions driving climate change, have little individual motivation to introduce regulation based on the idea of cost to mental health of inaction. Our results show that East Asia and the Pacific and South Asia incur the highest costs of mental ill health, and future global treaties and policies should reflect this.

Cost of illness assessment requires accurate information on medical costs, but accurate data are often missing, particularly in low- and middle-income countries. Conducting economic analysis that produces robust and nuanced results is technically challenging, and intangible social impacts are difficult to include, such as suffering and reduced quality of life.

In this case, it requires several inputs that are associated with uncertainties and debate. However, this should not preclude the fact that there is an obvious cost in terms of mental health due to environmental degradation.

The fifth United Nations Environment Assembly concluded with fourteen resolutions to strengthen action on nature to achieve the Sustainable Development Goals. Specifically, taking a One Health approach [66] (which includes human, animal and environment health as integrated and unified domains of programming) was highlighted as providing the strong political and scientific mandate to understand, identify and quantify the role of the planetary crisis on mental health. The Common Agenda of the Secretary General also provides the direction to identify the environmental dimension of the mental health [67].

## Additional Files

The additional files for this article can be found as follows:

10.5334/aogh.4079.s1 Appendix 1. Definitions and sources of data used to quantify relationships between the prevalence of mental illnesses and environmental factors and cost of mental illnesses and environmental factors.

10.5334/aogh.4079.s2 Appendix 2. Methodology for selecting and extracting data from the 24 meta-analyses included in the review of meta-analyses of environmental factors as determinants of mental health.

10.5334/aogh.4079.s3 Appendix 3. Economic scenario analysis results.

## Footnotes

DALY for a disease or health condition is a measure of overall disease burden, expressed as the number of years lost due to ill-health, disability or early death in a population.

GBD cause code B.6; cause ID 558.

The number of countries included in the analysis is limited to 170 due to an absence of data on the necessary explanatory variables for some countries.

Representative Concentration Pathway 4.5.
