# Supplementary material for: Planetary Health and Mental Health Nexus: Benefit of Environmental Management
Source: Ann Glob Health. 2023 Jul 24;89(1):49. doi: 10.5334/aogh.4079 (PMC10377746; doi:10.5334/aogh.4079)
Supplement: Appendix 1. — Definitions and sources of data used to quantify relationships between the prevalence of mental illnesses and environmental factors and cost of mental illnesses and environmental factors. [file agh-89-1-4079-s1.pdf]

Appendix 1: *Definitions and sources of data used to quantify relationships between the prevalence of mental illnesses and environmental factors and cost of mental illnesses and environmental factors*

Table A1.1. Definitions and sources of data used to quantify relationships at country level between the prevalence of mental illnesses and environmental factors

Variable	Definition/Proxy	Source	Mean	S.D.
**Rates of mental disorders**				
DALY rate	Number of mental health DALYs per 100,000 people (2019)	Global Burden of Disease	1,689.59	328.27
**Income**				
GDP per capita	Gross Domestic Product per capita (2019) (USD; thousands)	World Bank	17.34	24.04
**Within-country socio-economic variation**				
Income inequality	Index of income inequality (0–10)	INFORM Risk	4.04	1.95
Healthcare inaccessibility	Index of inaccessibility to healthcare (0–10)	INFORM Risk	4.13	2.45
**Environmental factors**				
Extreme natural disasters and climate-change events	Hazard Exposure Index – a composite index comprising data on hazards, exposure, vulnerability and coping capacity (0–10)	INFORM Risk	3.75	1.77
Air pollution	$PM_{2.5}$ mean annual exposure (micrograms per cubic meter)	World Bank	27.08	18.83
Access to open (green) space	Proportion of population living in urban areas (%)	World Bank	60.56	23.41

Table A1.2. Regression model for country-level analysis of rates of mental illness as a function of environmental, economic and socio-economic factors

	Regression coefficient (B)	Standard Error	Probability
Constant	1431.93	105.41	0.00
GDP per capita	**3.57**	1.12	0.00
Income inequality	**38.07**	14.98	0.01
Healthcare inaccessibility	**-94.06**	16.50	0.00
Extreme natural disasters and climate-change events	**42.68**	18.54	0.02
Air pollution	**2.12**	1.07	0.05
Access to open (green) space	**3.53**	1.10	0.00
N	170		
Adjusted R2	0.51		

Note: Dependent variable is the number of DALYs per 100,000 people

Table A1.3. Mean cost of mental illness or disorder by type of disorder

	Mean cost (US$/DALY)	Standard deviation	Sample size (N)
Common disorders	4,849	12,754	1,410
Severe disorders	11,399	20,787	465
Childhood disorders	62,071	189,655	273
Childhood + common disorders	1,120	872	62
Total	13,940	73,712	2,210

Table A1.4. Mean cost of mental disorder by type of cost

	Mean cost (US$/DALY)	Standard deviation	Sample size (N)
Healthcare costs	2,072	6,683	851
Productivity losses	9,210	21,832	208
Total societal costs	25,862	105,781	1,016
Other costs	7,036	45,653	149
Total	13,940	73,712	2,224

Table A1.5. Regression model for cost of mental disorders with

	Regression coefficient (B)	Standard Error	Probability (P)
Constant	-1.40	1.54	0.36
Severe disorders	**1.86**	0.13	0.00
Productivity costs	**1.76**	0.19	0.00
Societal costs	**2.56**	0.12	0.00
GDP per capita (ln)	**0.57**	0.14	0.00
Hazard exposure	**0.16**	0.03	0.00
Air pollution	-0.01	0.01	0.50
Accessibility to green space	0.00	0.01	0.47
N	2,086		
Adjusted R^2	0.23		

Note: Dependent variable is US$ per DALY at 2020 price level
Statistically significant relationships are given in bold
