# Supplementary material for: Planetary Health and Mental Health Nexus: Benefit of Environmental Management
Source: Ann Glob Health. 2023 Jul 24;89(1):49. doi: 10.5334/aogh.4079 (PMC10377746; doi:10.5334/aogh.4079)
Supplement: Appendix 2. — Methodology for selecting and extracting data from the 24 meta-analyses included in the review of meta-analyses of environmental factors as determinants of mental health. [file agh-89-1-4079-s2.pdf]

Appendix 2. Methodology for selecting and extracting data from the 24 meta-analyses included in the review of meta-analyses of environmental factors as determinants of mental health

We conducted systematic searches in three bibliographic databases: PubMed, PsycINFO and Embase. We first developed a general search string for climate change, pollution and green spaces, in which we combined text and key words for these subjects with text and keywords for mental health and mental disorders. We limited the results to systematic reviews and meta-analyses. Because the impact of climate events (such as tornados, landslides, heatwaves, etc.) may not be captured by keywords related to climate change, we conducted separate searches for these events. In these searches we combined text and key words for climate events with text and key words for mental health and mental disorders and again limited the results to meta-analyses and systematic reviews. The full search strings are available in Supplement 1. All records were read by two independent researchers, and we retrieved the full-text of all studies that were selected for retrieval by one or both researchers.

In this review we included meta-analyses that reported the association between (1) climate events, (2) green spaces or (3) pollution and mental health or mental disorders. We defined climate events as '*discrete episodes of extreme weather or unusual climate conditions, often associated with deleterious impacts on society or natural systems, defined using some metric to characterize either the meteorological characteristics of the event or the consequent impacts'* [68]. We used the taxonomy of climate events developed by Stephenson [69], including tropical cyclones, hurricanes, extratropical cyclones, convective phenomena (including tornadoes and severe thunderstorms), mesoscale phenomena (such as polar lows, resulting in for example extreme wind speeds and precipitation), floods, drought, heat waves, cold waves and fog. We also included natural events that may not be directly influences by the climate such as landslides. We included any kind of pollution, including specific substances, and pollution of air, soil and water. Any mental health problem or mental disorder was included. We excluded studies on the association between climate change and intelligence, as well as on dementia and cognitive decline. We decided to include only studies that

reported at least one analysis with 5 comparisons because this gives a reasonable impression of the association.

All full text papers were read by two independent researchers and the decision to include or exclude was based on consensus. Disagreements were solved through discussion. We extracted the following data from the included meta-analyses: the examined climate-related factor (climate event, pollution, specific substance, green spaces, etc.), the design of the included studies in the meta-analysis, the number of included studies, the aggregated number of participants in the primary studies (when reported), the population, and the instrument used to measure the quality of primary studies. We also extracted the mental health outcome, a summary of the pooled outcomes, the significance of the outcomes, the level of heterogeneity (I^2 and its 95% CI), and (when reported) the outcomes of the analyses examining publication bias. When I^2 or its 95% CI were not reported, we calculated them with the value of Chi-square and degrees of freedom (if available), using the Heterogi module in STATA SE (version 16.1 for Mac). The general characteristics of the meta-analyses were extracted by one reviewer. The outcomes were extracted by one reviewer whose extraction was validated by a second reviewer, who independently extracted 25% of the data. An agreement index of 96.3% between the two reviewers was reached.

References

68. Stott PA, Christidis N, Otto FEL, et al. Attribution of extreme weather and climate-related events. Wiley Interdiscip Rev Clim Change. 2016;7(1):23-41. doi:10.1002/wcc.380

69. Stephenson D. Definition, diagnosis, and origin of extreme weather and climate. In: Diaz HF, Murnane RJ, eds. Climate Extremes and Society. Cambridge University Press; 2008:11-24. https://www.cambridge.org/core/product/identifier/CBO9780511535840A009/type/book_part
