# Supplementary material for: Planetary Health and Mental Health Nexus: Benefit of Environmental Management
Source: Ann Glob Health. 2023 Jul 24;89(1):49. doi: 10.5334/aogh.4079 (PMC10377746; doi:10.5334/aogh.4079)
Supplement: Appendix 3. — Economic scenario analysis results. [file agh-89-1-4079-s3.pdf]

Appendix 3. Economic scenario analysis results

Table A3.1. Estimated prevalence (DALYs/100,000 people), cost (USD/DALY; 2020 price level) and change in annual total cost of mental disorders attributable to climate change, air pollution and access to green space (USD; 2020 price level; millions).

Country	DALYs per 100,000 people (2020)	Cost per DALY (2020) (USD)	Climate change cost (2030)	Air pollution cost (2030)	Green space cost (2030)	Climate change cost (2050)	Air pollution cost (2050)	Green space cost (2050)
Afghanistan	1,564	343	54.33	3.96	3.98	1,279.12	67.27	128.01
Albania	1,744	716	1.17	0.03	0.69	11.83	0.37	3.43
Algeria	1,769	1,444	84.71	29.01	17.39	902.23	244.68	138.41
Angola	1,372	533	22.14	0.37	6.93	330.45	1.67	74.74
Argentina	1,993	1,472	15.42	0.31	3.32	68.32	0.84	12.64
Armenia	1,787	1,380	4.27	0.22	0.50	5.02	0.73	2.63
Australia	2,033	1,988	26.94	0.19	3.35	157.88	0.65	20.61
Austria	1,855	1,628	3.65	0.13	1.83	15.99	0.53	9.91
Azerbaijan	1,810	1,378	32.19	0.78	4.39	621.52	10.56	68.06
Bahamas	1,871	1,553	0.54	0.01	0.04	2.07	0.03	0.23
Bangladesh	1,672	555	197.91	545.01	50.25	2,203.71	7,320.30	531.47
Belarus	1,783	1,234	5.04	0.16	1.97	21.64	0.68	6.17
Belgium	1,989	1,775	7.40	0.60	0.29	35.49	2.86	1.07
Benin	1,404	386	3.44	8.15	1.74	28.31	69.78	13.91
Bhutan	1,427	349	0.27	0.08	0.10	1.87	0.80	0.74
Bolivia	1,698	908	12.33	0.06	2.22	96.77	0.39	16.22
Bosnia and Herzegovina	1,557	1,101	4.20	0.10	0.91	29.54	0.92	6.95
Botswana	1,632	1,029	0.89	0.04	0.64	4.06	0.19	2.03
Brazil	1,971	1,875	244.83	3.26	44.74	1,496.20	13.56	211.28
Bulgaria	1,855	1,041	2.55	0.16	1.03	10.07	1.19	3.62
Burkina Faso	1,365	482	14.17	0.50	4.77	191.85	5.50	62.45
Burundi	1,305	335	9.57	0.19	1.14	170.02	3.08	23.52
Cambodia	1,449	646	18.14	0.75	3.66	176.83	4.63	44.97
Cameroon	1,611	775	34.66	4.40	8.25	423.91	28.88	88.99
Canada	1,937	2,206	60.54	0.45	5.38	354.90	2.08	43.68
Central African Republic	1,449	595	21.28	1.60	1.79	258.85	25.36	29.43
Chad	1,238	502	16.12	0.23	2.24	266.82	2.79	48.49
Chile	1,913	1,663	19.96	0.96	1.73	91.76	3.92	10.50
China	1,895	2,339	7,239.76	1,421.24	2,080.37	58,658.77	9,310.31	11,655.54

Colombia	1,957	1,699	120.24	0.62	14.88	1,144.34	3.51	112.03
Comoros	1,308	768	0.31	0.01	0.11	1.49	0.03	0.73
Congo, Democratic Republic of the	1,581	670	199.22	2.29	32.74	4,599.72	42.18	716.82
Congo, Rep. Of	1,541	638	3.38	0.06	1.01	37.20	0.55	9.35
Costa Rica	1,798	1,235	5.61	0.05	1.68	44.36	0.27	7.12
Cote d'Ivoire	1,484	663	29.56	0.80	6.23	362.98	8.02	78.16
Croatia	1,713	1,258	3.58	0.09	1.02	17.64	0.58	5.79
Cuba	2,107	1,429	12.38	0.18	1.16	63.04	0.85	8.75
Cyprus	1,694	1,179	1.26	0.07	0.09	8.01	0.41	1.15
Czech Republic	1,767	675	1.51	0.08	0.69	7.80	0.50	4.69
Denmark	1,921	1,391	0.90	0.05	0.42	3.55	0.19	1.71
Djibouti	1,518	726	1.35	0.06	0.10	9.56	0.53	0.92
Dominican Republic	1,684	947	8.67	0.11	2.80	63.04	0.67	11.99
Ecuador	1,718	848	26.69	0.10	2.38	253.20	0.61	31.93
Egypt	1,817	1,134	232.74	17.73	16.43	2,660.73	197.10	391.38
El Salvador	1,833	1,568	16.39	0.18	3.48	157.45	1.61	22.70
Estonia	1,651	627	0.14	0.00	0.08	0.63	0.02	0.41
Eswatini	1,342	504	0.13	0.01	0.06	0.64	0.05	0.34
Ethiopia	1,383	482	164.64	3.87	22.86	3,710.13	75.26	538.21
Fiji	1,529	985	0.29	0.00	0.19	1.63	0.01	0.85
Finland	1,902	1,567	0.60	0.02	0.36	2.21	0.08	1.84
France	1,952	1,887	34.27	1.28	14.23	161.31	5.86	56.60
Gabon	1,683	1,583	2.89	0.04	0.47	18.14	0.21	1.79
Gambia	1,370	454	0.76	0.07	0.41	4.75	0.45	2.25
Georgia	1,789	1,348	4.94	0.16	1.27	26.11	0.84	6.74
Germany	1,960	1,773	40.37	1.41	9.25	179.89	5.74	60.66
Ghana	1,479	409	9.63	2.42	4.84	88.79	15.38	37.65
Greece	1,965	1,628	15.88	0.39	2.67	98.99	3.23	13.90
Guatemala	1,643	1,129	41.86	0.63	5.95	466.25	4.99	70.68
Guinea	1,006	536	7.68	3.60	2.19	79.69	28.83	25.88
Guinea Bissau	1,299	362	0.90	0.05	0.21	5.41	0.46	1.73
Guyana	1,475	610	1.08	0.01	0.05	2.58	0.02	0.42
Haiti	1,369	622	12.51	0.11	3.73	214.26	1.24	37.00
Honduras	1,717	896	14.90	0.13	3.39	138.09	1.15	26.30
Hungary	1,793	874	3.90	0.11	1.34	21.14	0.75	7.42

Iceland	1,933	1,469	0.25	0.00	0.01	1.25	0.01	0.07
India	1,747	1,332	4,084.02	16,431.76	691.35	47,062.78	226,739.36	8,924.39
Indonesia	1,567	1,532	726.64	28.74	175.54	5,607.77	151.63	1,259.20
Iran, Islamic Republic of	1,995	2,014	276.78	139.66	44.71	2,373.64	1,414.65	302.13
Iraq	1,826	2,432	239.06	10.59	19.10	3,911.42	193.70	350.38
Ireland	1,838	1,596	1.33	0.04	0.99	5.69	0.14	5.00
Israel	2,152	2,880	29.45	9.50	1.32	164.63	53.73	7.55
Italy	2,027	2,193	87.80	1.81	19.16	452.70	11.55	99.06
Jamaica	1,591	1,214	1.99	0.04	0.71	18.85	0.18	5.13
Japan	2,103	2,955	316.63	5.02	16.11	1,533.08	20.45	83.49
Jordan	1,777	632	4.23	1.80	0.63	31.63	12.74	3.22
Kazakhstan	1,611	905	12.85	0.38	2.07	80.85	2.21	19.59
Kenya	1,405	693	69.14	1.59	13.92	787.21	19.03	176.25
Kuwait	2,180	1,591	1.50	0.62	-	5.67	2.99	-
Kyrgyzstan	1,602	1,467	81.24	0.90	4.56	705.41	10.63	74.16
Laos	1,357	505	3.51	0.34	1.49	28.49	2.41	12.27
Lativa	1,707	1,115	(0.41)	0.02	0.13	0.12	0.06	0.64
Lebanon	1,865	1,414	15.39	15.64	1.02	109.06	114.90	6.45
Lesotho	1,296	631	0.67	0.09	0.35	3.73	0.49	2.06
Liberia	1,253	303	0.67	0.02	0.46	8.52	0.13	4.38
Libya	2,071	5,022	79.61	1.34	6.58	731.69	15.24	51.80
Lithuania	1,770	1,071	0.90	0.03	0.36	3.90	0.13	1.84
Luxembourg	1,955	1,668	0.13	0.01	0.07	0.56	0.03	0.21
Madagascar	1,310	425	15.53	0.15	5.70	212.27	1.25	73.00
Malawi	1,269	355	7.04	0.10	1.55	74.69	0.64	21.74
Malaysia	1,788	1,494	41.97	1.16	11.63	251.56	5.53	53.41
Mali	1,394	756	43.62	0.56	9.73	937.79	9.46	174.89
Mauritania	1,384	791	6.70	0.06	1.90	71.75	0.72	16.47
Mexico	2,069	2,847	522.60	9.92	59.55	4,387.40	60.33	437.29
Moldova	1,565	1,428	3.24	0.06	0.54	17.96	0.38	4.80
Mongolia	1,663	521	0.75	0.14	0.18	4.98	0.83	1.73
Morocco	1,699	1,107	47.35	6.28	12.32	358.48	45.63	82.61
Mozambique	1,430	280	12.84	0.10	3.35	213.76	0.99	52.25
Myanmar, Union of	1,545	2,031	373.50	33.46	35.24	4,274.66	349.29	585.22
Namibia	1,742	1,033	1.77	0.03	1.18	10.64	0.11	5.12
Nepal	1,466	306	18.40	44.69	2.97	258.35	711.85	49.28
Netherlands	1,944	1,587	4.71	0.46	2.79	19.29	1.92	6.42

New Zealand	1,943	1,631	4.06	0.02	0.44	22.79	0.06	2.95
Nicaragua	1,686	470	4.22	0.03	0.62	44.07	0.27	7.97
Niger	1,452	719	48.49	0.83	3.19	1,224.55	13.63	137.78
Nigeria	1,394	925	715.17	31.37	127.80	15,117.43	292.59	2,173.70
North Macedonia	1,669	769	2.55	0.05	0.41	24.87	0.77	4.11
Norway	1,921	1,531	0.71	0.02	1.00	2.98	0.07	3.03
Oman	1,973	1,736	6.45	28.34	2.52	39.62	180.40	7.70
Pakistan	1,615	670	562.15	84.08	47.67	12,527.93	1,622.34	1,478.69
Palestine	1,984	973	3.40	1.74	0.79	22.28	11.11	4.88
Panama	1,818	1,252	10.28	0.04	1.31	59.10	0.29	9.34
Papua New Guinea	1,310	1,131	22.51	0.06	1.38	198.75	0.39	26.34
Paraguay	1,651	894	4.34	0.03	1.16	29.03	0.13	8.56
Peru	1,759	1,520	46.68	0.54	6.14	294.38	3.67	43.64
Philippines	1,698	2,206	530.96	5.97	60.03	4,971.02	43.76	803.04
Poland	1,620	626	7.24	0.16	1.80	42.04	1.49	23.51
Portugal	1,786	1,227	5.11	0.07	2.82	25.56	0.34	11.36
Qatar	2,260	935	0.42	0.63	0.03	1.50	2.38	0.06
Romania	1,636	926	11.11	0.27	2.05	61.68	2.50	19.12
Russian Fed.	1,966	2,415	656.40	5.80	50.49	5,720.00	64.71	518.31
Rwanda	1,370	437	5.93	0.38	0.85	58.57	2.23	14.31
Saudi Arabia	2,230	2,261	71.08	9.70	9.59	471.35	69.00	56.79
Senegal	1,456	562	8.89	0.59	3.03	87.53	4.66	30.71
Serbia	1,828	2,059	19.87	1.72	2.16	158.91	12.06	22.05
Sierra Leone	1,199	230	1.44	0.10	0.52	16.40	0.90	6.10
Slovakia	1,631	790	2.44	0.06	0.42	12.01	0.36	4.82
Slovenia	1,659	1,230	1.55	0.03	0.44	9.07	0.23	2.88
Solomon Islands	1,305	582	0.28	0.00	0.12	2.39	0.01	0.90
South Africa	1,769	1,553	112.87	5.11	23.84	777.90	33.42	139.38
South Korea	1,933	1,862	79.63	20.40	3.17	457.91	90.72	47.25
Spain	2,009	1,690	40.68	0.73	8.93	203.77	3.74	41.22
Sri Lanka	1,489	471	11.27	0.29	1.53	77.24	1.88	20.29
Sudan	1,610	899	114.89	3.42	14.22	2,225.12	68.99	350.23
Suriname	1,610	1,436	0.50	0.01	0.06	1.93	0.03	0.44
Sweden	1,961	1,572	1.65	0.04	1.46	6.72	0.17	4.27
Switzerland	1,939	1,822	4.56	0.10	0.94	20.36	0.42	6.87
Syrian Arab Republic	1,701	2,310	136.78	14.91	21.05	2,343.18	246.03	309.46

Tajikistan	1,624	716	27.45	1.09	2.18	1,343.95	37.33	117.81
Tanzania	1,373	221	19.14	0.29	5.95	224.34	3.33	64.87
Thailand	1,695	2,117	224.13	16.16	58.49	1,541.24	82.93	362.79
Togo	1,392	480	3.12	8.93	1.45	22.84	54.03	10.73
Trinidad and Tobago	1,793	1,522	1.06	0.02	0.15	4.05	0.08	1.19
Tunisia	1,723	1,339	14.29	5.24	3.04	95.76	31.49	20.55
Turkey	1,986	1,834	243.31	27.71	40.20	2,112.01	217.74	270.74
Uganda	1,447	511	53.28	2.41	11.32	1,131.23	41.68	216.65
Ukraine	1,626	1,535	164.05	1.69	9.65	2,139.81	27.28	168.41
United Arab Emirates	1,961	1,308	5.60	1.13	1.28	23.69	4.98	4.32
United Kingdom	1,997	2,164	77.60	1.23	15.70	394.98	6.54	69.48
United States of America	2,227	4,393	2,361.04	10.06	184.89	22,257.36	82.59	1,554.21
Uruguay	1,926	1,115	0.35	0.01	0.13	1.26	0.03	0.37
Uzbekistan	1,694	966	52.45	3.42	2.95	679.04	37.37	88.30
Venezuela	1,772	1,401	41.47	0.40	1.73	317.84	2.44	20.00
Vietnam	1,577	807	129.73	132.79	38.82	1,238.64	855.76	357.82
Yemen	1,664	1,580	244.09	18.08	30.66	6,972.51	528.46	806.22
Zambia	1,450	406	5.97	0.15	2.83	46.72	0.72	21.93
Zimbabwe	1,337	652	10.35	0.09	1.23	81.74	0.76	21.49
Global			23,247.96	19,255.39	4,380.31	245,281.72	252,273.37	39,029.02
